# The root cause analysis on failed patient‐specific measurements of pencil beam scanning protons using a 2D detection array with finite size ionization chambers

**DOI:** 10.1002/acm2.13343

**Published:** 2021-07-26

**Authors:** Jacob C. Ricci, Wen C. Hsi, Zhong Su, Karl Mund, Robert Dawson, Daniel J. Indelicato

**Affiliations:** ^1^ Department of Radiation Oncology University of Florida College of Medicine Gainesville FL USA; ^2^ Department of Radiation Oncology University of Florida Health Proton Therapy Institute Jacksonville FL USA; ^3^ Department of Medical Physics University of Florida College of Medicine Gainesville FL USA; ^4^ Department of Radiation Oncology Ackerman Cancer Center Jacksonville FL USA; ^5^ Present address: Department of Radiation Oncology Orlando Health UF Health Cancer Center Ocoee FL USA

**Keywords:** 3D gamma index analysis, detector effects, patient‐specific measurements, proton therapy, quality assurance

## Abstract

The aim of this report is to present the root cause analysis on failed patient‐specific quality assurance (QA) measurements of pencil beam scanning (PBS) protons; referred to as PBS‐QA measurement. A criterion to fail a PBS‐QA measurement is having a <95% passing rate in a 3.0%‐3.0 mm gamma index analysis. Clinically, we use a two‐dimensional (2D) gamma index analysis to obtain the passing rate. The IBA MatriXX PT 2D detection array with finite size ionization chamber was utilized. A total of 2488 measurements performed in our PBS beamline were cataloged. The percentage of measurements for the sites of head/neck, breast, prostate, and other are 53.3%, 22.7%, 10.5%, and 13.5%, respectively. The measurements with a passing rate of 100 to >94%, 94 to >88%, and <88% were 93.6%, 5.6%, and 0.8%, respectively. The percentage of failed measurements with a <95% passing rate was 10.9%. After removed the user errors of either re‐measurement or re‐analysis, 8.1% became acceptable. We observed a feature of >3% per mm dose gradient with respect to depth on the failed measurements. We utilized a 2D/three‐dimensional (3D) gamma index analysis toolkit to investigate the effect of depth dose gradient. By utilizing this 3D toolkit, 43.1% of the failed measurements were improved. A feature among measurements that remained sub‐optimal after re‐analysis was a sharp >3% per mm lateral dose gradient that may not be well handled using the detector size of 5.0 mm in‐diameter. An analysis of the sampling of finite size detectors using one‐dimensional (1D) error function showed a large dose deviation at locations of low‐dose areas between two high‐dose plateaus. User error, large depth dose gradient, and the effect of detector size are identified as root causes. With the mitigation of the root causes, the goals of patient‐specific QA, specifically detecting actual deviation of beam delivery or identifying limitations of the dose calculation algorithm of the treatment planning system, can be directly related to failure of the PBS‐QA measurements.

## 1 INTRODUCTION

Patient‐specific quality assurance is a necessary step to detect actual deviation of beam delivery or limitations of dose calculation algorithm in a treatment planning system (TPS) for radiation cancer treatments using either external particle or high‐energy photon beams. While pencil beam scanning methods have been widely adopted in centers worldwide due to the ability to produce excellent dose conformity,^1^ the variations in dose from spot to spot and from layer to layer necessitates an accurate and reliable method of ensuring agreement between the delivered and planned doses.^2^ As technologies continue to develop, the relative errors associated with each aspect of a particle therapy system consistently decrease. However, there are still factors of a treatment plan that can lead to dose or spatial deviations.^3^ Moreover, the quality assurance (QA) process itself can introduce errors between planned and delivered doses.^4^ While accurate device setup is crucial in any radiotherapy measurement process, it is drastically more important in measurements of highly modulated therapy modalities as multiple measured points over a variable plane must agree with planned values to conclude accurate machine delivery.

Multiple styles of two‐dimensional (2D) detector arrays are utilized in the measurement of highly modulated fields. However, in pencil beam scanning (PBS) and other particle‐based modalities, ion chamber arrays, such as the IBA MatriXX (IBA Dosimetry, Schwarzenbruck, Germany) are common and normally paired with other devices to facilitate measurement of beams at multiple depths. However, one notable shortcoming of these detectors is that they only measure 2D planar doses. When utilizing these devices to measure beams that are highly modulated with respect to depth, as is the case for most proton therapy beams, small deviations in depth can lead to large deviations between measured and planned doses.^5^ To this point, there have been studies analyzing the role of machine parameters^3^ as well as detector choice in proton therapy QA results.^6^ However, in‐depth analyses of the errors causing suboptimal QA results and methods to mitigate those errors are not common. While PBS presents a benefit in the realm of dose conformity and healthy tissue sparing, the high degree of modulation present requires highly accurate measurements to determine accuracy of the delivered beam.^7^ Any associated errors with this measurement setup, if not handled appropriately, could cause possible delivery discrepancies to go unnoticed. Therefore, an investigation into the common errors associated with the QA process in modulated PBS plans can provide a useful guide to determine whether any future QA results below a passing threshold are related to the deviations of actual beam delivery, the limitation of TPS calculation, or the setup errors of using a specific detection system.

In this study, we present a root cause analysis on the patient‐specific measurements using PBS protons; referred to as PBS‐QA measurement. Failed PBS‐QA measurements over a period of 2 years at our institute were analyzed to mitigate the errors that arise. Of the 2488 PBS‐QA measurements, only 159 have failed the initial QA test indicating that there is generally good agreement between planned and measured doses. Analysis of those failed PBS‐QA measurements revealed three root causes that likely point to failure: (1) incorrect measurement device set up, (2) measurement within a high‐dose gradient with respect to depth, and (3) a combined effect of detector size and spacing within the plane of measurements. Errors in the comparison for the first two causes may be reduced through re‐measurement or shifting measurement results within a QA program and utilizing a 2D‐to‐three‐dimensional (3D) γ‐index analysis toolkit, respectively. Errors introduced by the third cause are more difficult to reduce and would require more precise simulation with analytical parameters to be introduced upon commissioning of the treatment machine as well as improved measurement hardware. Overall, this analysis was conducted to provide additional methods testing to better determine if a low passing rate was the result of error within the QA process or a true discrepancy between the plan and delivered doses.

## 2 MATERIALS AND METHODS

### 2.1 The QA procedure with the selection of measurement depths

The PBS delivery system was implemented in the second gantry room at our institute beginning in May 2017. We describe the characteristics of PBS beam delivery and the commissioning of TPS^8^ in a separate section. Based on the database of recorded PBS‐QA measurements, the numbers of patients and of PBS‐QA measurements are sorted and listed in Table 1. The patients with head/neck, breast, or prostate cancer were the major disease sites treated using PBS protons. In total, 404 patients and the 2488 measurements were utilized to conduct the root cause analysis of the failed PBS‐QA measurements in this study. Because treated patients usually had a complicated target shape, we used multiple‐field optimization in the fashion of intensity‐modulated proton therapy (IMPT) for all of patients using PBS protons. Additionally, since the majority of patients are treated with a 74 mm range shifter, we evaluate each PBS‐QA measurement by comparing it to a 2D dose distribution at the measured depth of each field calculated using a Monte Carlo (MC) algorithm in water tank with a gantry angle of 270°.

**TABLE 1:** Distribution of patients and quality assurance (QA) measurements in 6‐month intervals for the pencil beam scanning (PBS) system at our institute

Site	Period	2017: Oct–Dec 2018: Jan–Jun	2018: Jul–Dec	2019: Jan–Jun	2019: Jul–Dec
All	Patients	220	83	73	28
	Measurements	974	455	800	259
Head/Neck	Patients	102	32	31	8
	Measurements	498	250	460	118
Breast	Patients	37	15	24	3
	Measurements	200	83	192	90
Prostate	Patients	13	4	8	9
	Measurements	122	34	80	24
Other	Patients	68	32	10	8
	Measurements	154	88	68	27

Note

The total numbers for all of disease site are listed at the top two columns. Total numbers of 404 patients and the 2488 measurements were utilized to conduct the root cause analysis of the failed PBS‐QA measurements. All disease sites that were not located in the specified areas were grouped into the “Other Category.”

The commercially available 2D ionization array IBA MatriXX PT^9^, ^10^ is used for planar dose measurements. The MatriXX PT array consists of 1020 air‐vented plane‐parallel ionization chambers in a 32 × 32 matrix with a sampling distance of 7.62 mm over an area of 244 mm × 244 mm. Each chamber has a 4.2 mm in‐diameter with a height of 2.0 mm. The effective measurement point is at 6.0 mm below its outside cover. Depth adjustments for each measurement depth are made using a DigiPhant (IBA Dosimetry, Schwarzenbruck, Germany) water phantom. To accommodate the non‐flat holder of MatriXX PT, a ~9 cm front wall extension was added at the top of DigiPhant. Therefore, utilizing a 30 × 40 cm range shifter restricts certain snout positions due to this front wall extension. This results in some QA measurements that utilize a range shifter being performed with larger air gaps than planned. When the air gap difference is less <3.0 cm, most measurements still passed a 3%/3 mm γ‐index analysis. However, failures were observed for measurements with more than a 4.0 cm difference from the planned air gap. Once the failure was observed to be associated with an incorrect location of range shifter, measurements were repeated with solid water blocks allowing the planned air gap to be met.

**FIGURE 1:**
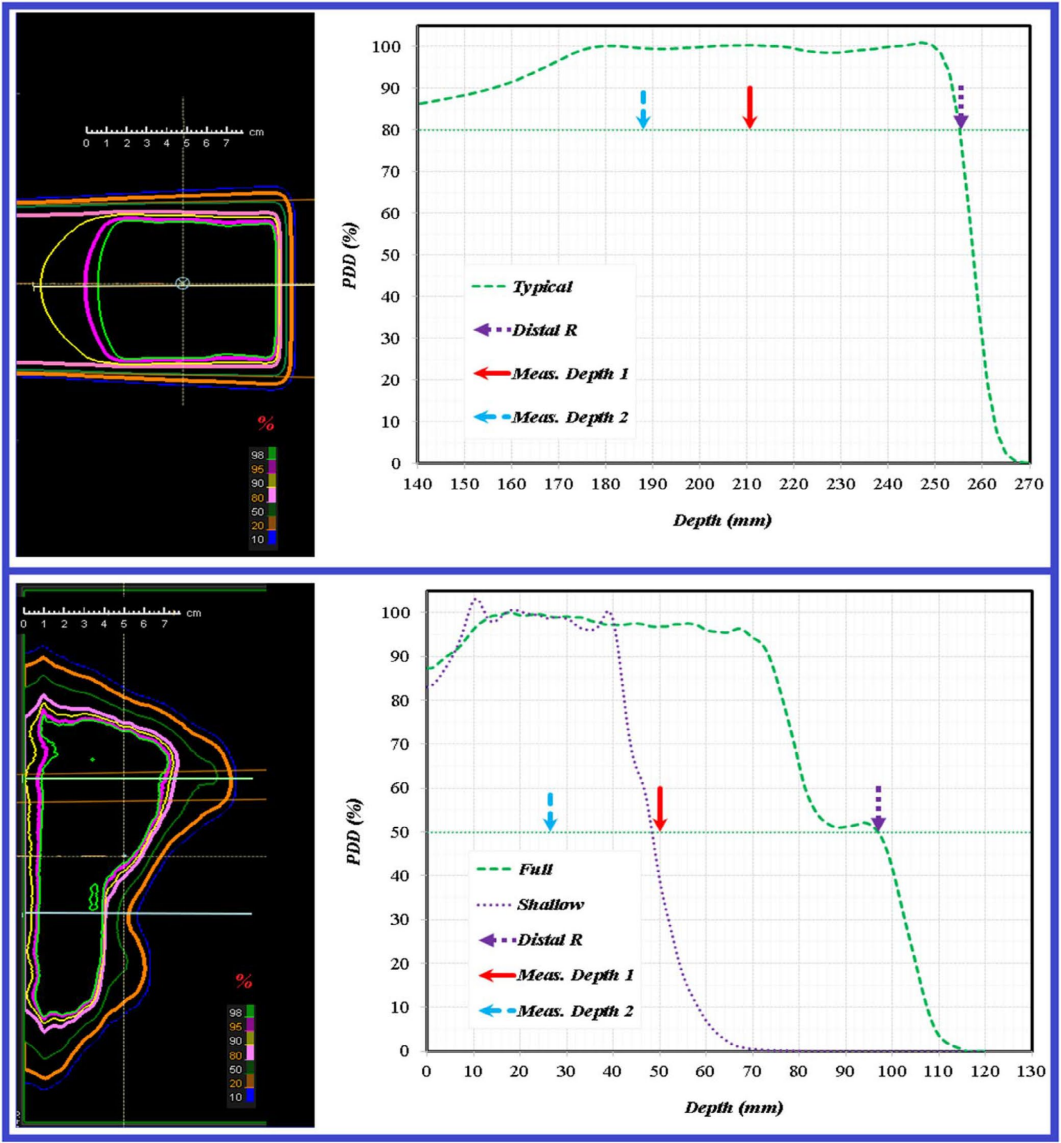
Depth dose distributions for an ideal (top) and real (bottom) proton beam. The standard criteria of measurement depth selection for each field is shown for both cases. The depths selected are one‐half and one‐quarter of maximum penetration range. In the real case, this depth selection can lead to measurement in areas of high‐dose gradients with respect to depth as is indicated by the purple dashed line

The standard procedure for each treatment field requires measurements at two depths. The selection of depths of a field is presented in an ideal and a real situation as shown at top and bottom panels in Figure 1, respectively. For ideal situations, the measurements would be performed at depths of one‐half and one‐quarter of penetration range over a uniform modulated dose distribution. Clinically, there were no measured beams, among the analyzed set, that had an ideal uniform dose distribution. For the real situation, measurement depths being one‐half and one‐quarter of maximum penetration range can result in a depth dose gradient of >3% per mm in some areas of measured planar dose distributions as shown in the right bottom panel of Figure 1. Each 2D measured planar distribution was analyzed with respect to planned 2D distribution through a 2D 3%/3 mm γ‐index analysis in the IBA myQA (IBA Dosimetry, Schwarzenbruck, Germany) platform.

At our institute, a measurement with <95% γ‐index passing rate is considered a “failed measurement.” Of the root causes for the failed PBS‐QA measurements listed in the introduction, the error of “incorrect measurement device set up” is referred as a “user error” in this study. The user error can be classified into (1) incorrect use of a beamline device such as a shifter as “improper beamline device‐IBD” error. (2) Misalignment of the MatriXX PT or the isocenter location in water phantom as “Alignment of measurement equipment‐AME” error. (3) Incorrect association of depth between calculated and measured planar doses as “Improper Planner Dose –IPD.” When a measurement fails in the normal workflow, the first check confirms the accuracy of the detector setup while the second check is to review the calculated 2D dose distribution at the requested depth in the corresponding QA plan. In parallel, one re‐measurement is performed to rule out any malfunctioned detector array and/or a beam delivery error. Failed measurements with passing rate of <80% were strongly related to the setup error. We further investigated retrospectively failed measurements with a passing rate between 80% and 95% for the root causes.

### 2.2 The commissioning of TPS for a PBS beam delivery system

We carefully investigated the beamline characteristics of the PBS delivery during the commissioning after the hardware upgrade from double scattering delivery in our gantry room 2. The investigated beamline characteristics included, but were not limited to, the variation of absolute dose delivery in terms of gantry angle or field size, the variation of source‐to‐axis distance between various energies, and the in‐air spot size/shape and its alignment for each energy layer. Overall, this beamline can produce beams with a distal 80% range (R80) between 46.4 and 324.4 mm corresponding to energies of 75.19 and 227.48 MeV, respectively. In total, there are 151 possible energy layers available for treatments and there is a 0.31–3.84 mm water equivalent thickness (WET) difference between neighboring energy layers. However, due to spot sizes exceeding clinically acceptable limits, the lowest energy used for treatments is 99.59 MeV which sets a minimum range limit at 76.6 mm. A total of 141 energy layers are used for treatments. The spot sizes along the X/Y axes at isocenter are 12.05/11.99 and 5.00/5.04 mm for lowest, 99.59 and highest, 227.48 MeV, energies, respectively. The minimum and maximum deliverable MU for each spot are 0.1 and 12.0 MU. The dose rate of each spot was dynamically adjusted to not only minimize its delivery time but also to allow enough time to shut the beam off and to avoid saturating the maximum charge rate of monitor chamber (518 nA). The switch time between the energy layers is ~7.0 s due to slow controllers of the magnets installed 15 years ago. The upgraded switching time will be within 3.5 s within 1 year.

We used RayStation (RS) TPS^11^, ^12^, ^13^ (RaySearch Laboratories, Stockholm, Sweden), version 6.1, for the PBS treatment planning. Because of the long switching time, we do not use the PBS protons for cancers in the thorax or abdomen with a large (>5.0 mm) target motion. We did not study the interplay effect^14^ during the commissioning of our TPS. We collected the beam data required to commission this TPS for both analytical PB and MC dose algorithms. Because the energy layer of this beamline is discrete instead of continuous as described above, we correctly listed the 141 discrete energy layers in the general tag of the pencil beam scanning data of the TPS for energies between 99.5 and 227.48 MeV. We used a Bragg Peak plane‐parallel ionization chamber with 81.6 mm diameter to measure the integrated depth dose (referred to as IDD_Mea) of each energy layer for all 141 energy layers, but only imported 30 of IDD_Mea with a ~10 mm WET difference between imported energy layers. We validated the correction of proton fluence loss for 30 IDDs imported into the TPS.

For the treatment of a target at a shallow depth less than ~70.0 mm, a flat range shifter is used to produce sufficient doses at the proximal edge of target. A shallow depth target typically occurred for patients with cancer of the breast, head/neck, or prostate with pelvic nodes. We also carefully studied the dosimetric deviations induced using the range shifter. The vendor provided only one Lexan shifter with a nominal physical thickness of 65.0 mm and a WET of 74.0 mm for the 400 mm × 300 mm snout. In addition, we fabricated Lucite range shifters for the snouts having a circular open of 250, 180, and 100 mm. We optimized each in‐house range shifter to match the WET pullback within +/−0.2 mm with respect to the vendor's range shifter. The modeled pullback of the range shifter in RS TPS was on average 0.3 mm smaller (range: −0.2 to −0.5 mm) than measured pullback. We validated the 3D dose calculations with the range shifter at various air gaps and energy layers with both the PB and MC algorithms in RS TPS. We present the details for the validations of the TPS commissioning and the effect of range shifter in the result section.

### 2.3 The 3D γ‐index toolkit to re‐analyze failed measurements

The effect of a >3% per mm depth dose gradient was observed on many failed measurements during the 2D γ‐index re‐analysis using myQA. To properly take the effect of high depth dose gradient into account, an in‐house 2D/3D γ‐index analysis toolkit was built in MATLAB (MathWorks, Natick, MA). We conducted the data analysis using the 2D/3D toolkit retrospectively, because the in‐house analysis toolkit was not available during measurements for this study. This 2D/3D toolkit utilizes a similar conceptual approach as a previously published program.^15^ Calculated 3D dose distribution with a dose grid of 2.0 mm after reading into the 3D toolkit was re‐binned to have a 1.0 mm pixel resolution. The 2D dose distribution of each measurement was properly aligned within 1.0 mm to the calculated 3D distribution. A global threshold of 10% of maximum dose was applied to exclude measured points having lower dose level. Two arrays with a voxel size of 1.0 mm are then created. In the first array, voxels at or below the specified search radius contain the associated linear distance values from the center point. The second array is a binary copy of the first and serves as the search matrix. The 3D dose distribution matrix is multiplied by the search matrix to give the calculated dose values within the search radius for a given measured point. Then, it calculates γ‐index values for each point only over the search matrix instead of over the whole volume of 3D dose distribution to speed up the γ‐index analysis. The γ‐index values were calculated as specified in Low et al^16^ and were stored in a separate array. Once calculations for all included measurement points were completed, the percentage of passing points was returned for the passing of each measurement.

### 2.4 Effects of finite size ionization chamber and the spacing between neighboring chambers

An ideal 2D array should have small‐sized chambers with little spacing between neighboring chambers for measurement. However, the signal noise acquired by small volume ionization chamber can be at a comparable level to the signal itself. Therefore, we investigated the effect of the detector size and the sampling distance of MatriXX PT for failed measurements, that did not improve after the 2D/3D γ‐index re‐analysis. The extracted one‐dimensional (1D) dose profiles from the calculated 2D planar dose distribution through an area of highest γ‐index values were compared with the corresponding measured 1D doses in a column or row of MatriXX PT array. Additionally, simulated profiles generated by an error function were compared with measured and calculated profiles to determine a root cause of failed measurements.

**FIGURE 2:**
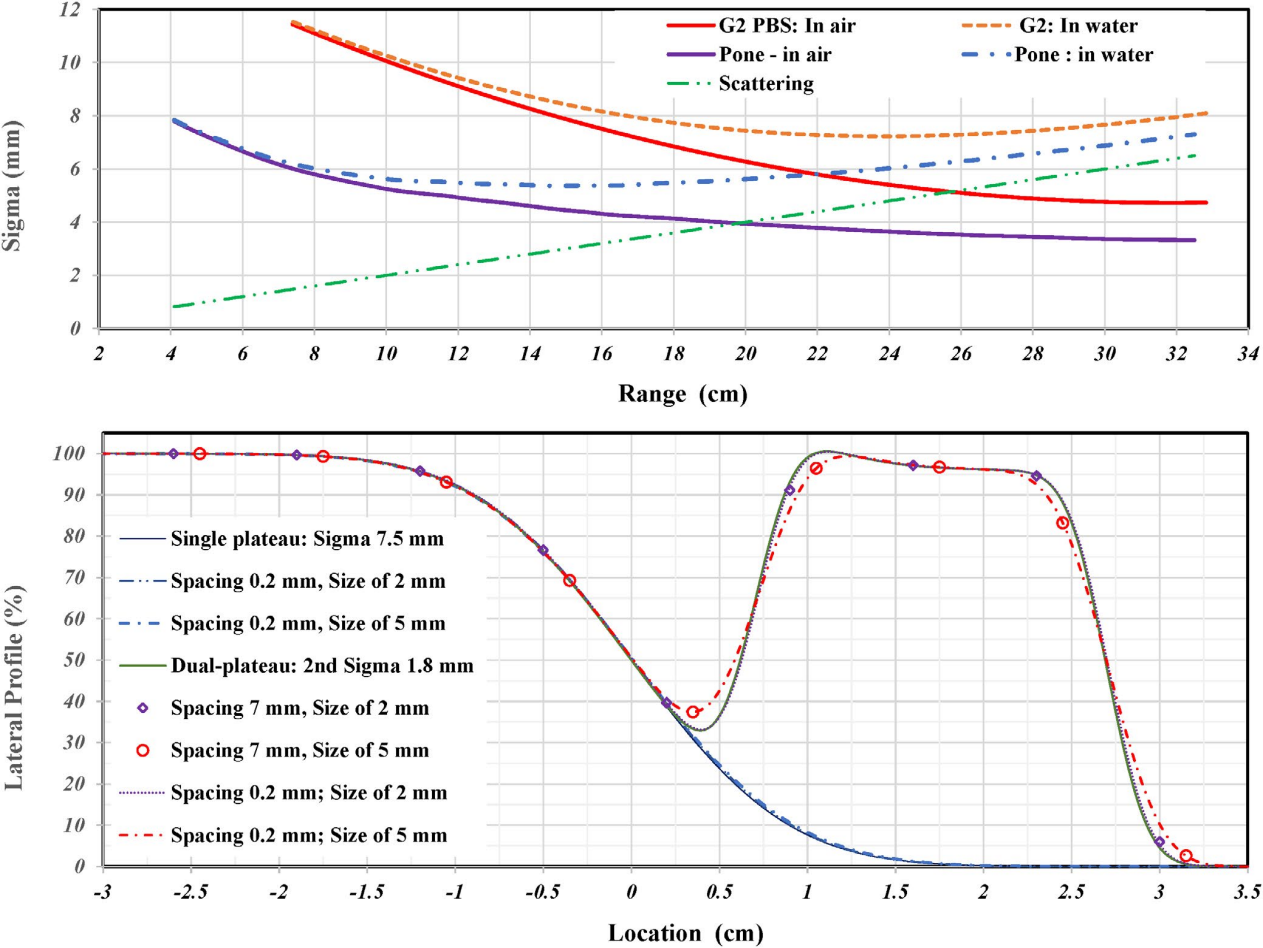
Top: Solid lines present the spot size in air of the G2 (IBA Proteus+) and P#x02010;one (IBA Proteus One) as a function of beam range. The linear increasing of scattering for proton passing water over a length of beam range. Shown dashed lines are the spot size in water; it includes both effects of the scattering and the spot size in air. Bottom: An error function with specified sigma for each plateau was used to simulate a 1D single‐ or dual‐plateaus lateral profile. A sigma of 7.5 mm was used to generate the single plateau and the first peak of dual‐plateaus profile. To consider a high‐dose gradient shaped by a sharp distal penumbra along depth of beam path, a sigma of 1.8 mm was used for second peak of dual‐plateaus profile. The average dose within detectors with diameters of 2.0 and 5.0 cm and a spacing of 7.0 mm was calculated to present the effects of finite size detector and its spacing in used 2D array in this manuscript

To select the proper sigma to simulate the lateral profile, the in‐air spot size as a function of range was studied and plotted solid lines in the top panel of Figure 2 for the G2 PBS beamline of our proton multiple room system (IBA Proteus+) and the P‐One beamline in our proton one room system (IBA Proteus One). The PBS‐QA measurements used in this study were performed in our multiple room system. Besides the characteristics of spot size in air, scattering increases linearly as penetration depth increases. The increasing of spot size in water is linear as shown by the dashed straight line at top panel of Figure 2. By combining the spot size in air and the scattering in water, the spot sizes in water at the depth of beam range are plotted as dashed lines for the G2 and P‐One beamline at top panel of Figure 2. Without a range shifter, a minimum sigma of ~7.5 cm in water at a range of ~22 cm is seen for the G2 beamline while a minimum sigma of ~5.5 cm in water at a range of ~12 cm is seen for the P‐One beamline. Therefore, a sigma of 7.5 mm for PBS‐QA measurements performed in the G2 beamline was used to simulate the single peak and the first peak of dual‐plateau lateral profile as solid line curves at bottom panel of Figure 2. The penumbra of lateral profile was calculated by an error function using the assigned sigma. To present a high‐dose gradient shaped by a sharp distal penumbra along depth of beam path, a sigma of 1.8 mm was used for the second peak of dual‐plateaus profile. When the dose gradient at the shoulder of signal‐plateau profile is between 3% and 5% per mm, there is minimal variation due to averaging in detector volumes with diameters of 2 or 5 mm as shown in the three overlapped curves in the bottom panel of Figure 2. For a large dose gradient of ~10% per mm at the shoulder of second peak of dual‐plateau lateral profile, a small variation (<2%) for a chamber size of 2 mm is shown as the diamond points in the bottom panel of Figure 2. However, a large deviation (>5%) between the original profile and the averaged measurement with a chamber diameter of 5 mm was seen and is shown as circles in the bottom panel of Figure 2. The variation (~8%) is more prominent in the low‐dose region between two plateaus with higher doses due to the averaging with higher doses near the lowest point of valley.

## 3 RESULTS

### 3.1 The passing rate statistics of γ‐index analysis of PBS‐QA measurements

The numbers of measurements for “All Sites” and disease sites of head/neck, breast, prostate, and other are listed over various γ‐index intervals in Table 2. The percentage of measurements performed for each site of head/neck, breast, prostate, and other are 53.3%, 22.7%, 10.5%, and 13.5%, respectively. The percentage of all measurements, that is, “All Sites,” for the γ‐index intervals 100 to>94%, 94 to>88%, and <88% are 93.6%, 5.6%, and 0.8%, respectively.

**TABLE 2:** Distribution of measurements over various intervals of γ‐index passing rates and anatomical site

Rate Site	100 to >97	97 to >94	94 to >91	91 to >88	88 to >85	85 to >82	82 to >80	<=80
All Sites	1876	453	99	41	12	2	2	3
Head/Neck	1042	219	45	17	3	0	0	0
Breast	378	131	27	20	4	2	2	1
Prostate	210	38	7	1	2	0	0	2
Other	246	65	20	3	3	0	0	0

Note

The number of measurements within a specific range of γ‐index interval is displayed. The total number of measurements analyzed was 2488.

Of the 2488 measurements taken at the time of this study, only 159 failed to pass initial testing. Upon analysis of these failed measurements, 13 improved upon re‐analysis using the 2D γ‐index analysis in the commercial myQA platform, 69 improved to passing levels upon re‐analysis using the house‐build 2D/3D γ‐index analysis toolkit, and 23 were likely attributable to the effects of detector size or spacing resolution. Fifty‐four measurements were no longer accessible for this study due to a change in planning system and a lack of plan data for these beams. The root causes for failed measurements described above are further investigated below.

### 3.2 User Errors during measurements and data analysis

**FIGURE 3:**
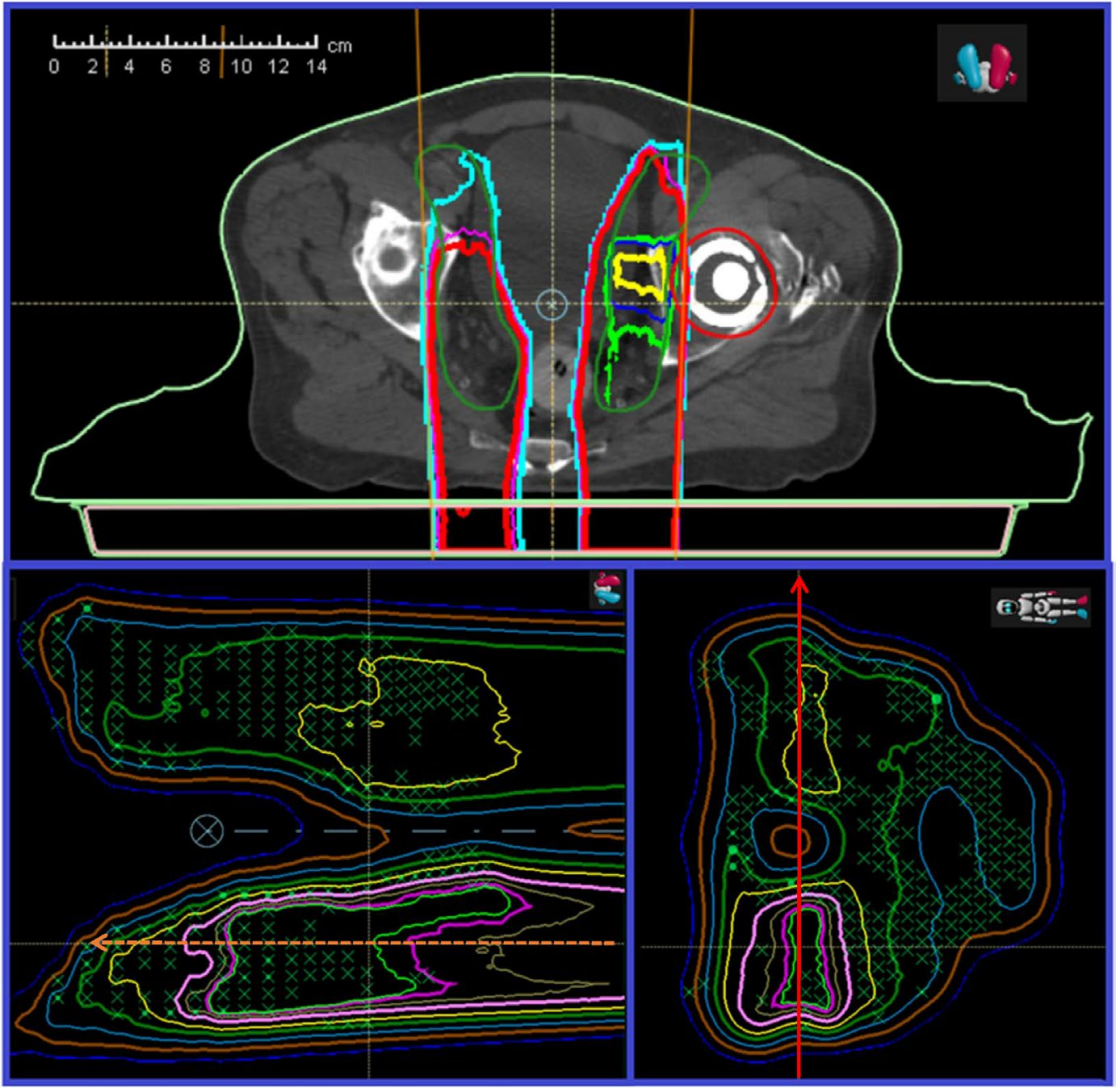
Clinical plan and quality assurance (QA) calculation of prostate case with artificial hip. Top: A posterior–anterior (PA) field with a 74 mm range shifter was used to treat the prostate cancer with pelvic nodes of a patient with a metal hip replacement. Bottom Left: Shows calculated two‐dimensional (2D) dose distributions in water along the beam path for QA measurements. Bottom Right: The calculated 2D dose distributions in water at a depth perpendicular to the beam path. The noticeable difference between the two sides of the PA field stems from the presence of the artificial hip. This difference would be made up for with a lateral beam on the patient's right side but this is not feasible on the left side

User error was the cause of 13 failed measurements which improved upon 2D γ‐index re‐analysis. Due to an improper beamline device—IBD error, 3 out of 13 failed measurements, 2 in a prostate plan and 1 in a breast plan, initially failed with rates below 80%, by placing a 7.4 cm range shifter for prostate cases requiring no range shifter, and by not placing a 7.4 cm range shifter for breast case that required it. Besides the misuse of the range shifter, there was an air gap 6.0 cm larger than the planned due to the missing range shifter. The larger air gap is also an IBD error. For a clinical example, the top panel of Figure 3 shows the failed measurement of a posterior–anterior (PA) field used to treat a prostate cancer with pelvic nodes for a patient with a metal hip replacement. Two distinguished dose levels between two lateral nodes are seen because the posterior field provided a full dose to the side with the hip replacement while this PA field and a lateral field on the opposing side of the patient equally contribute to the dose. A small 6.0 cm air gap was used to achieve a sharp lateral dose gradient in the treatment plan. However, an air gap of >12.0 cm results in low γ‐index passing rates of ~90% for measurements of the PA field at two depths. Repeated measurements of the PA fields using solid water block and a 6.0 cm air gap had γ‐index passing rates well above the 95% threshold.

**FIGURE 4:**
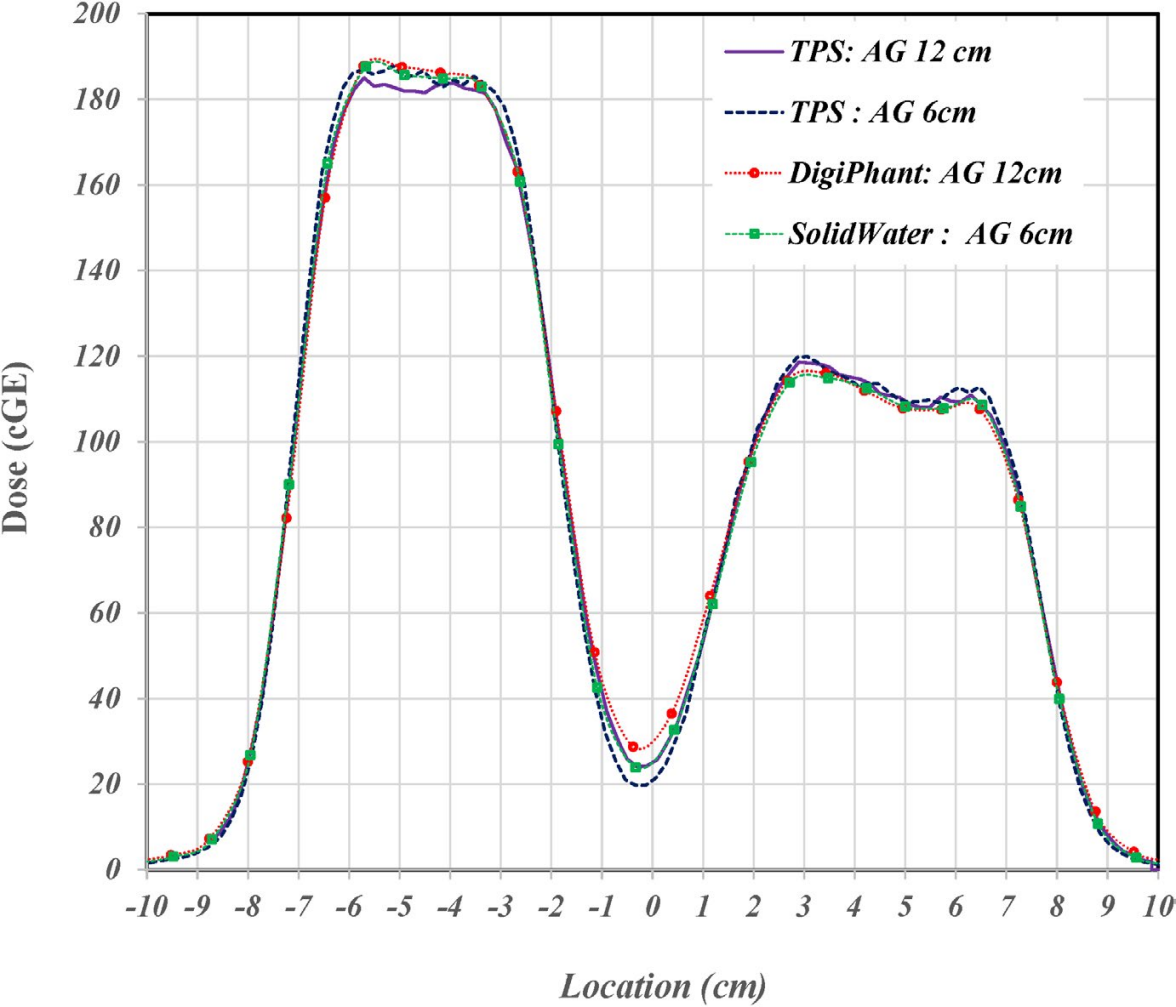
Lateral dose profiles and measurements using different air gap distances. The extracted lateral profiles are at the 10 cm depth from the calculated 3D dose distributions following the solid arrow indicated in Figure 3 with an air gap (AG) of 6 and 12 cm as the dashed and solid lines, respectively. The location of extraction was selected to match one row of detectors in measurements. Measured lateral profiles using the DigiPhant with a 12 cm AG and using solid‐water with a 6 cm AG at same location are plotted by circle and rectangular points, respectively

To investigate the effect of the 6 cm air gap difference, the 3D dose calculations for air gaps of 6 and 12 cm were recalculated with the MC algorithm. Bottom panels of Figure 3 show 2D dose distributions with a 12 cm air gap along and perpendicular to the beam path. The lateral 1D profiles passing two distinguished dose levels as indicated arrow at the bottom right panel of Figure 3 were extracted. Figure 4 shows extracted lateral profiles for air gaps of 6 and 12 cm with solid and dashed lines, respectively. Measured lateral profiles using the DigiPhant with a 12 cm air gap and a 6 cm air gap at the same location were extracted and plotted in Figure 4 by circular and rectangular points, respectively. Differences between calculated lateral profiles for the air gaps of 6 and 12 cm were found to be ~5% at the valley between two plateaus, and ~3% at the shoulders of each plateau. Measurement at a depth of 10 cm using the DigiPhant with an air gap of 12 cm is ~8% higher at the valley in comparison to the calculated distribution of a 6 cm air gap. This difference is reduced to ~4% in comparison to the calculated distributions of air gap of 12 cm. Measurement at a depth of 10 cm using solid water with an air gap of 6 cm has only a ~3% different at the valley to the calculated distribution of air gap of 6 cm. A >3% difference for measured doses using either the DigiPhant or solid water were found in the valley between two plateaus.

In addition to IBD errors, an error of misalignment between measurement and planned conditions, referred to as an AME error, can result in a sub‐optimal γ‐index passing rate. To avoid any significant misalignments, care is taken to align the device as accurately as possible. X‐Ray images are taken before a reference field is delivered. However, the setup of isocenter to a depth is manually performed with an associated error of +/−1.0 mm. To alleviate an AME error, multiple measurements with depths of both proximal and distal were performed, but steep dose gradients along depth can hinder this benefit as noted earlier.

Besides IBD and AME errors, the planar 2D dose distributions from the planning system could be exported at the wrong depths as required in the QA form. We refer to this as an IPD error. When the γ‐index analysis in myQA of a measurement is suboptimal, the user is allowed to choose the calculated dose distribution at a depth within ±1.0 mm of measurement condition because the resolution along the depth of QA field is 2.0 mm in the TPS. However, this shift in the depth helped in only a few cases. The majority of the 13 failed measurements required a re‐exported and re‐import of the planar doses to achieve >95% pass rate with a 3%/3 mm of γ‐index criterion.

### 3.3 The validation of the commissioned TPS

**FIGURE 5:**
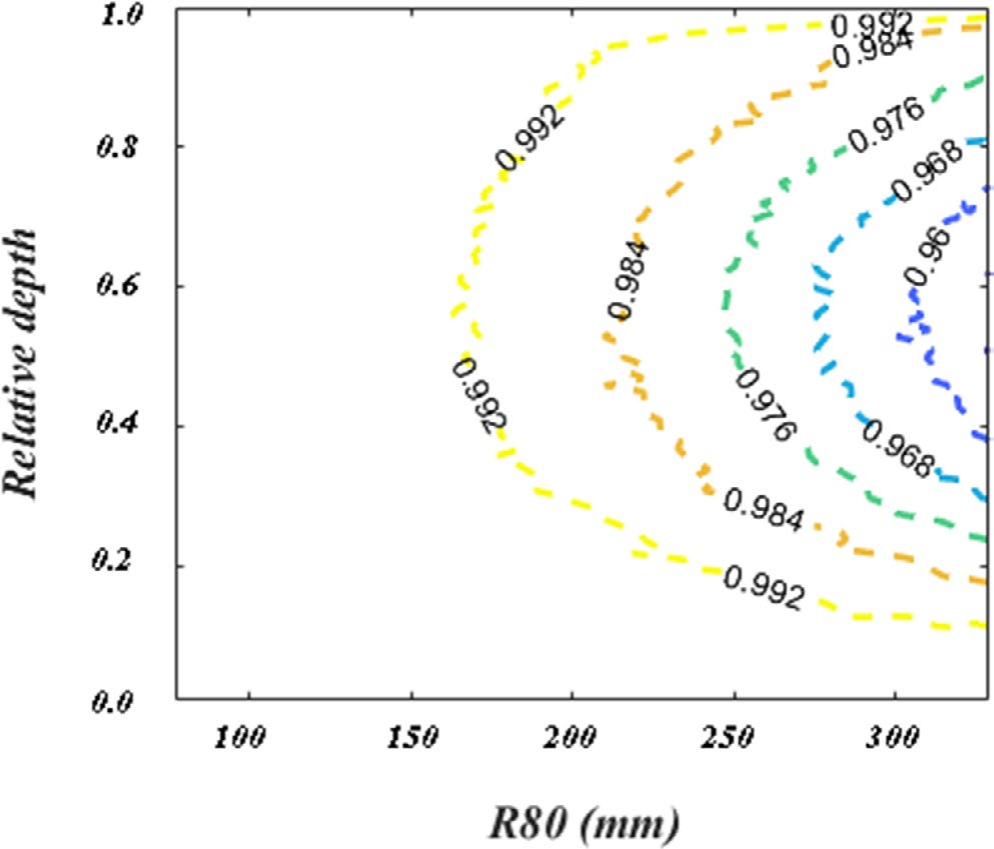
Geant4 calculated fraction of the IDD_Mea fluence measured by an 8.2‐cm diameter detector as function of R80 and relative depth (i.e., measurement depth divided by R80)

To validate the range and shape for the 30 IDDs imported into the TPS, we calculated 3D dose distribution of each energy layer with a 120 mm × 120 mm uniform spot pattern at each energy layer. These layers were summed over the whole calculation plane at each depth to generate a calculated IDD (referred as IDD_Cal). We generated an IDD_Cal using both PB and MC dose algorithms. The comparison between IDD_Mea and IDD_Cal of each energy showed a variation of less than 0.7 mm in the R80 ranges for all 30 energy layers. However, because the size of used Bragg peak chamber was not large enough to collect all large angle scattered protons, we observed a ~4% deviation between IDD_Mea and IDD_Cal due to the fluence loss at depths equal to half the range for IDDs with largest beam range. The RS TPS uses Monte Carlo simulations to calculate the fluence loss at each measured IDDs according to the size of chamber. An interpolated parameterization of fluence loss was used to correct IDD_Mea in RS TPS. To quantitatively study the effect of fluence loss, we also performed in‐house Geant4 simulations^17^ of mono‐energetic pencil beams to present the fraction fluence measured by an 81.6 mm diameter chamber versus the full IDDs in the 2D maps of measured R80 range and the relative depth (i.e., Measurement depth divided by R80). The results are shown in Figure 5. With the correction, we observed small deviations, less than 1.0% and 0.5% for IDD_Cal using MC and PB algorithms, respectively, at all depths except near the pristine peak.

To validate the depth dose using the range shifter in RS, we used both PB and MC algorithm to calculate the 3D dose distributions of a 100 mm × 100 mm uniform spot pattern.

**FIGURE 6:**
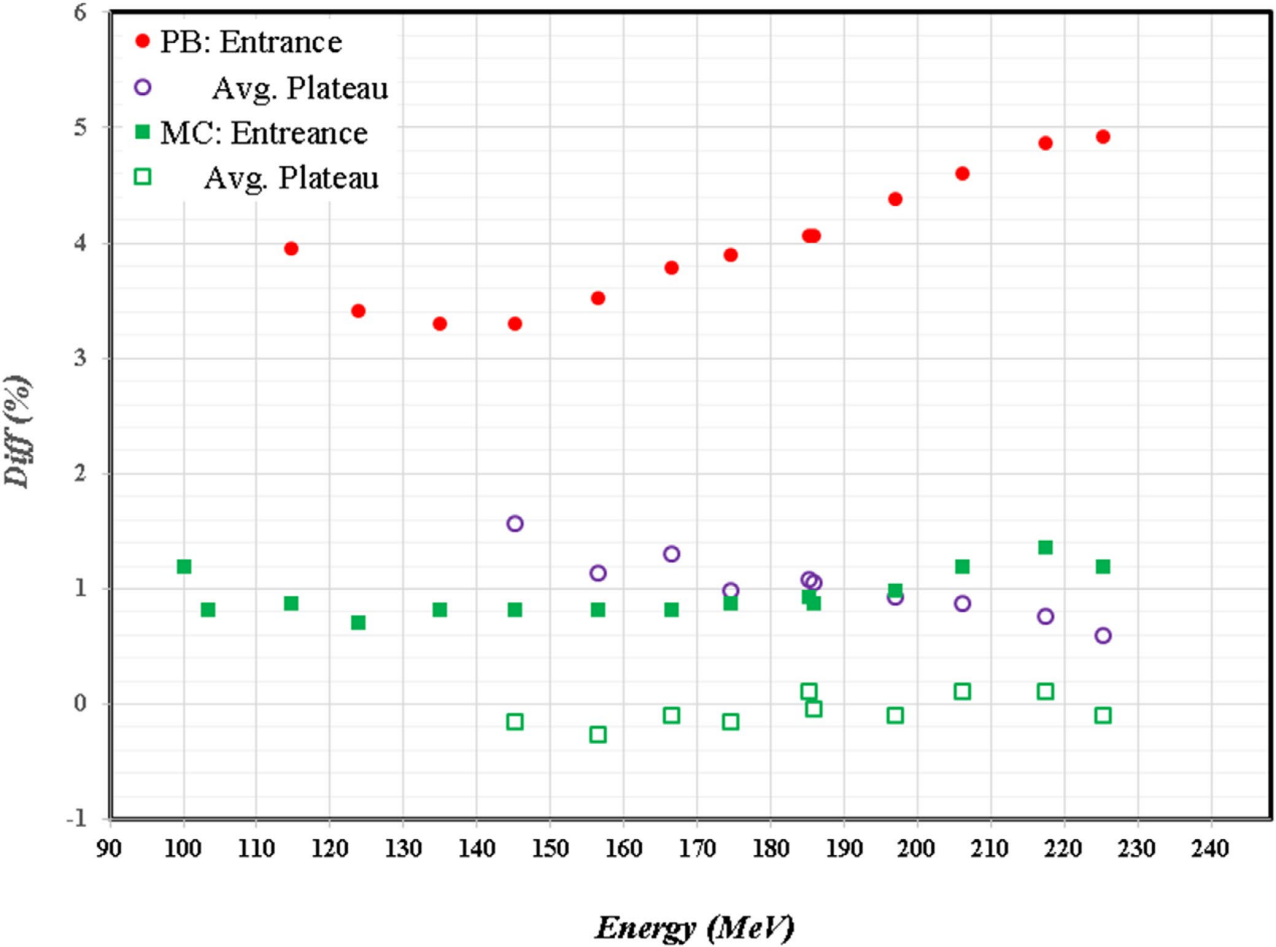
Percentage deviations between the PPD_Cal of PB/MC and PDD_Mea at the depth of entrance and the averaging over depths at the plateau as a function of beam energy for an air gap of 180 mm. Solid/opened circles are for PPB_Cal of pencil beam algorithm, and solid/opened squares are for PPB_Cal of Monte Carlo algorithm.

Then, we obtained the percentage depth dose (PDD_Cal) by averaging over a 20 mm × 20 mm area at the central axis for each depth. We measured the PPD (PDD_Mea) using a multi‐layer ionization chamber^18^ (MLIC) with a 1‐inch in‐diameter chamber at each layer. We applied the proper correction of the SAD effect of PPD_Mea using MLIC with average density less than water. To study the difference between PPD_Cal and PPD_Mea, each PDD was normalized to its peak for obtaining the “absolute” percentage difference. When comparing a PDD_Mea with a PPD_Cal using PB algorithm with 90.0 mm air gap behind the 74.0 mm range shifter, the PPD_Cal (PB) of 205.16 MeV protons is 3.5% higher at the entrance than PPD_Mea, and reduces to 0.5% at a depth of 50.0 mm. Larger doses up to 5% were present at entrance for PPD_Cal(PB) for the 330.0 mm air gap. However, the PDD_Cal (MC) using MC algorithm is within 1.0% in comparison with PDD_Mea for air gaps from 90.0 to 330.0 mm. Figure 6 shows the trend of percentage deviation between the PPD_Cal of PB/MC and PDD_Mea as a function of beam energy. Because about half of patients treated in our GTR2 used the range shifter, we used the MC algorithm to calculate the 3D doses in a QA water phantom to avoid the induced variation of PB algorithm for the γ‐index analysis of all PBS_QA measurements.

### 3.4 The validation of the 2D/3D γ‐index analysis toolkit

**FIGURE 7:**
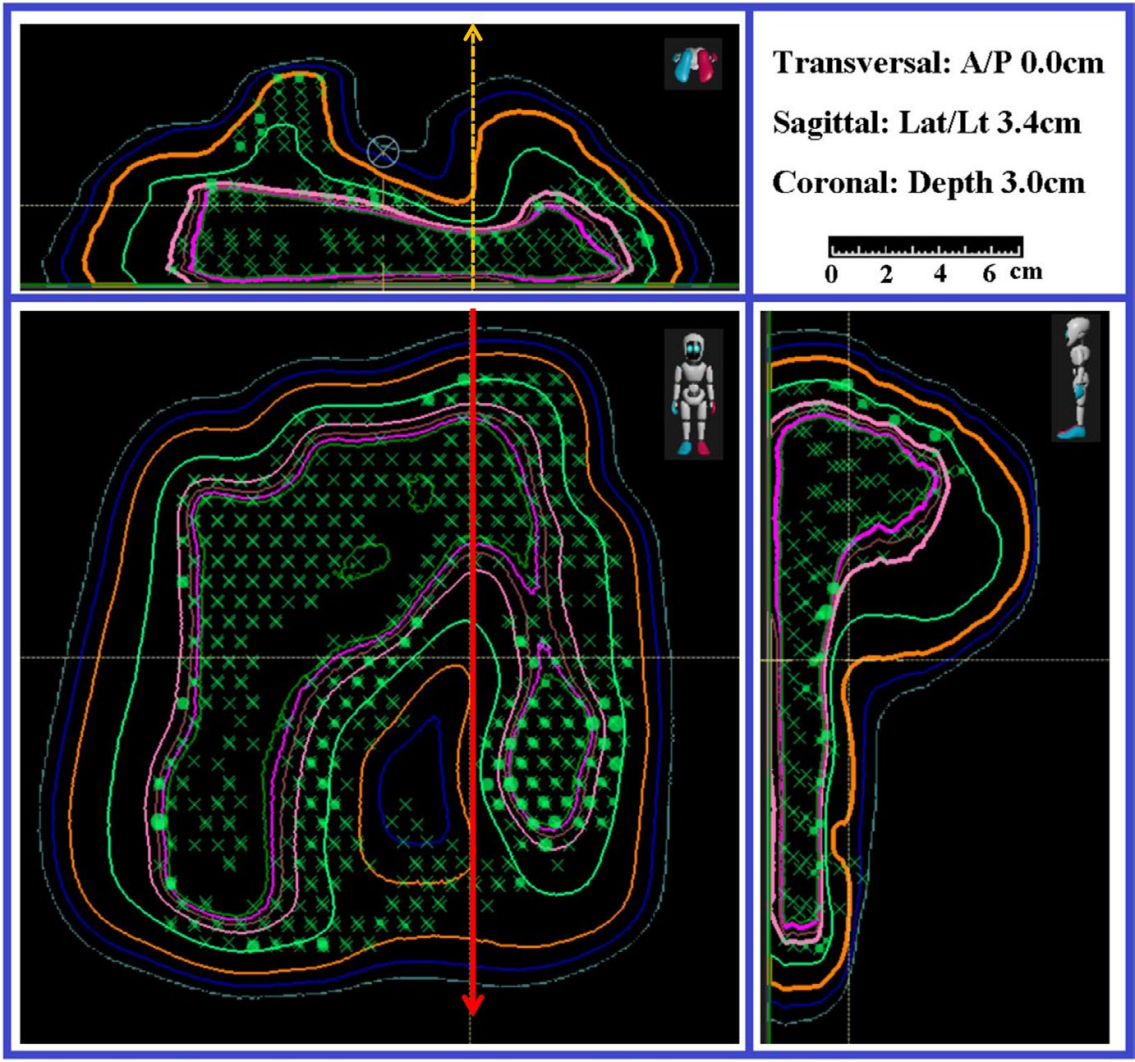
Isodose and spot pattern distribution in three planes at requested depth of measurement. This Figure shows the three cut‐planes of two‐dimensional (2D) dose distribution of a typical field in breast cases. The location of three cut‐plan is indicated at top right corner. Measurement depth is at 3 cm. Measured 2D dose distribution will be compared with calculated one at bottom left. One depth dose and one lateral profile were extracted at the locations of dashed and solid arrows, respectively

**FIGURE 8:**
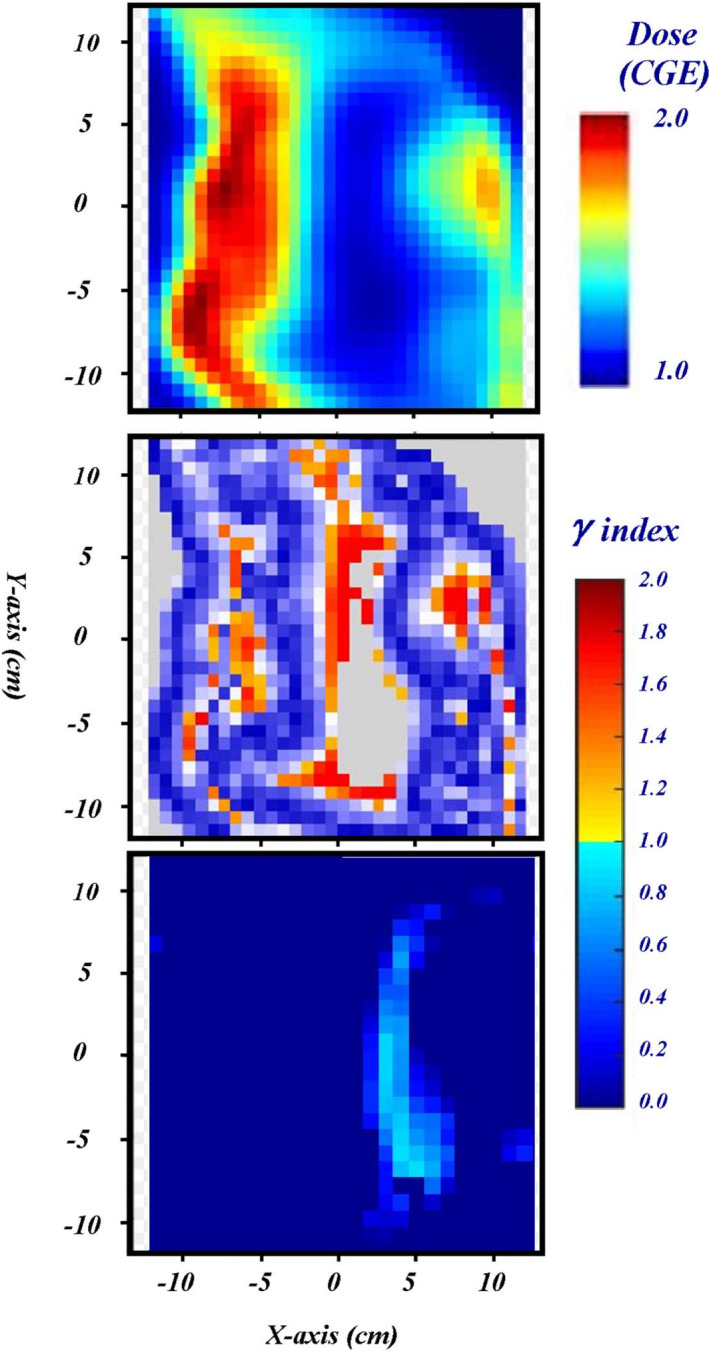
Gamma map output of a quality assurance (QA) beam using two‐dimensional (2D) 3%/3 mm analysis and 2D/3D 3%/3 mm analysis Top: A measurement of dose distribution of an oblique field for a breast case. This is the measured distribution from the beam in the bottom left panel in Figure 4. Middle: Shows the obtained γ‐index map of this measurement using 2D γ‐index toolkit in myQA platform. Bottom: Shows the obtained γ‐index map by house‐built using 2D/3D γ‐index toolkit using criteria of 3%/3 mm

Utilization of the 2D/3D gamma index showed a drastic improvement in the results of 43.1% of beams that initially failed QA testing. The majority of improvement was seen in the breast plans as shown in Figure 7, but this may be due to their large makeup of the original sample, that is, the majority of failed measurements were in breast plans. To show an example of the noticeable improvement, measured 2D distribution for a left anterior oblique field of a breast case is shown at the top panel of Figure 8. The distribution of 2D γ‐index values for the measurement in Figure 8 is shown in the middle panel. The area of gray color indicates the measured doses below a set 10% global threshold. The red‐like points indicated values that are above 1.0 and are in the valley between two high‐dose regions, in the highly varied dose gradients at the shoulders of plateau, or near the peak dose areas. The passing rate of γ‐index for this measurement using 2D γ‐index data analysis was 86.7%. Reanalyzing this data using the 2D/3D γ‐index toolkit produced the distribution shown in the bottom panel of Figure 8. A γ‐index criteria of 3%/3 mm was used in both γ‐index data analyses. In the 2D/3D γ‐index analysis, many points that previously failed now returned values well under 1.0. The passing rate of γ‐index for this measurement using 2D/3D γ‐index data analysis with a global threshold of 10% was improved to 99.8%. This large change is due to the inclusion of a third dimension along the depth in the search function.

**FIGURE 9:**
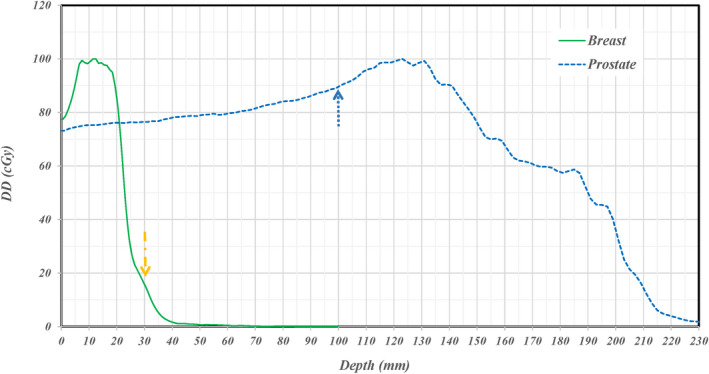
Extracted depth doses in areas of high gamma values for two clinical cases. Extracted depth doses along the dashed arrows indicated in Figures 5 and 7 for prostate and breast cases are shown. The dose gradient along depth is >6.0% per mm at the depth of measurement for the breast case and is <2.0% per mm at the depth of measurement for the prostate case

Breast cases that specified measurement depths <35 mm required the use of solid water plates. However, the usage of solid water plates does not lead the overall failure of the measurements. The other cases that benefitted from using the 2D/3D γ‐index were not necessarily subject to the user error. The subsequent case is specifically selected to note the potential of this program to correct for both small depth errors with the high depth dose gradient (DDG) as well as larger errors due to user error. For an in‐depth analysis of the plan itself for breast and prostate cases, depth doses were extracted and are plotted in the Figure 9 with the arrows specifying requested measurement depths in Figures 3 and 7. For the depth doses passing the high‐dose region at the depth of 10 cm in the prostate plan, the DDG is <2% of its maximum dose per mm (2%/mm). The small DDG does not cause a measurement failure with solid water for the prostate cases. For the depth doses passing the area of high γ‐index values in the measurement of breast case, a high DDG of >6%/mm in combining with a misalignment can cause the failure of measurement in the 2D γ‐index data analysis. The effect of high DDG was included when using the 2D/3D γ‐index data analysis.

### 3.5 Effects of sampling spacing and size of ionization chamber

**FIGURE 10:**
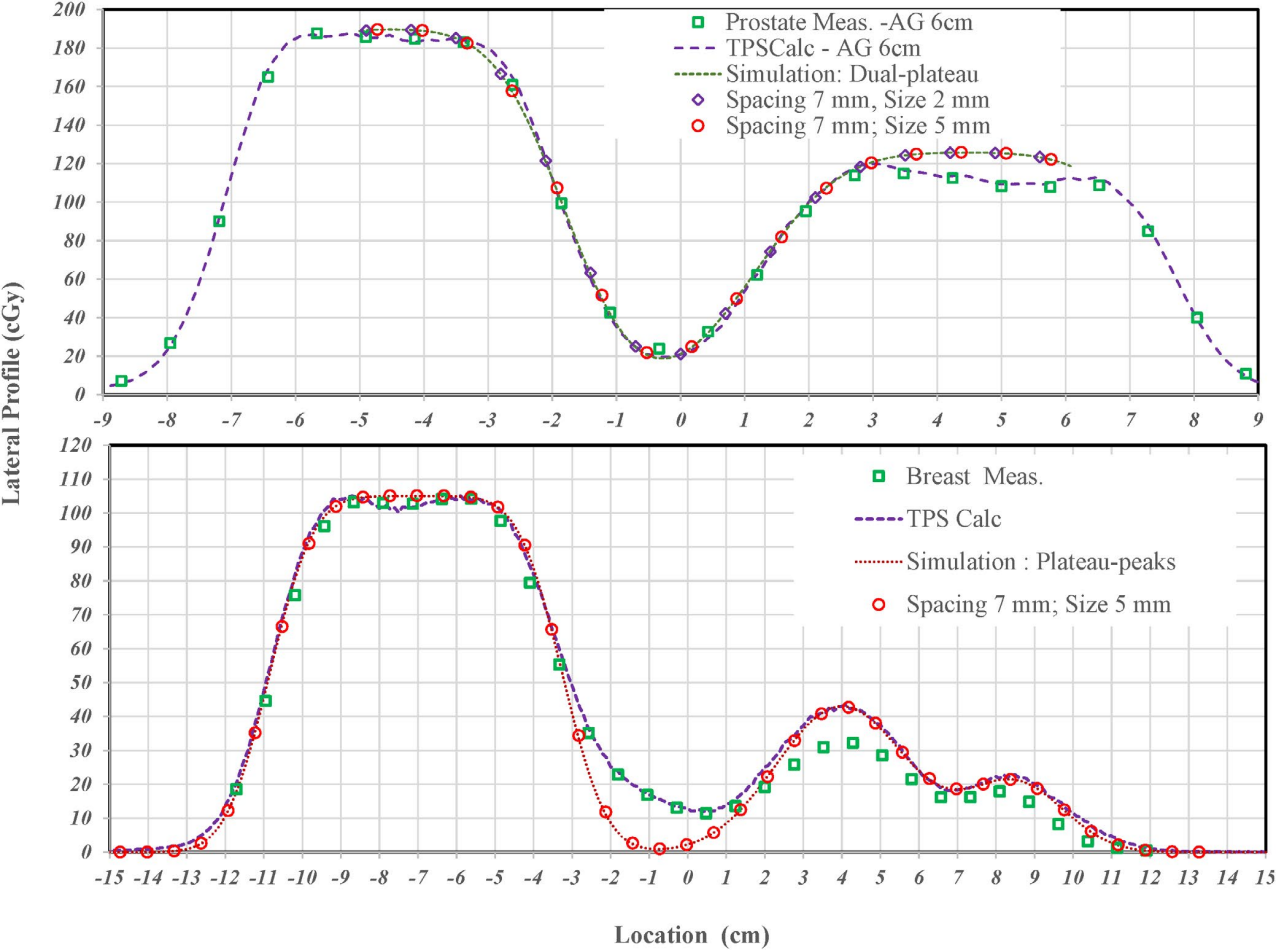
Comparison of lateral dose profiles extracted from TPS and measurements to an analytical simulation to examine possible detector effects. Square points are for measured lateral profiles for prostate and breast cases at top and bottom panels, respectively. Extracted TPSCalc lateral profiles are presented by dashed curves while the analytical simulated profiles are presented by dotted curves. Diamond and circle points, respectively, present effects for detector sizes of 2.0 and 5.0 mm with a spacing of 7.0 mm

In the analysis of the failed QA beams, 14% present likely detector effects that cause sub‐optimal pass rates. As with the 2D/3D gamma script, two examples are shown to illustrate the noticeable deviation between measured and exported. When these cases were analyzed in the 2D/3D gamma script there was only moderate improvement signifying that the errors were not in the proximal/distal direction. The 1D lateral profiles; referred to as TPSCalc, were extracted from TPS calculated 2D dose distribution along the direction indicated by an arrow in Figure 3 for a prostate case and in Figure 7 for a breast case. The lateral profiles pass the high‐dose region of two plateaus for the prostate case and intersects in the areas of the highest gamma values for the breast case. The location of each extracted lateral profile was aligned to the location of a column of ion chamber array in each measurement. Each extracted profile from the TPS calculation is plotted by a dashed curve and the corresponding measurement by square points at top and bottom panel of Figure 10 for the prostate and breast cases, respectively.

For the prostate case, points of measurements agree well with the TPSCalc curve except for points of measurement in the valley between two plateaus where there is a ~2.5% deviation. By properly matching the TPSCalc curve for the prostate case to the analytical simulation as shown dotted curve at the top panel, the sigma was found to be 8.7 and 10.6 mm for first and second plateaus, respectively. The large sigma of each plateaus indicates a dose gradient of <2.0%/mm. So, the effect of detector size is minimal for of the 2 and 5 mm diameter detectors as shown by diamond and circle points, respectively, when these points follow the simulated curve. The result indicates that the 1D analytical simulation needs to be further improved for the complex dose shaping in a 2D lateral dimension and/or a 3D dimension including depth.

For the breast case, points of measurements only agree well with the TPSCalc curve around the large plateau but are about 3% lower in second and third peaks. This clearly shows an under‐response in areas that correspond to the original failures from the initial 2D γ‐index analysis. To simulate the TPSCalc curve, the sigma for the plateau and each peak was found to be 9.0, 14.0, and 11.0 mm, respectively. However, the complex dose shaping between the plateau and the first peak by the depth dose gradient could not be correctly simulated with the simple error function. Similar to the prostate case, the large sigma indicates a minimal effect of detector size as shown circle points for a size of 5 mm when the circle points follow the simulated curve. Based on the 1D analytical simulation for both prostate and breast cases, the effect of detector size in one dimension of a 2D lateral profile was found not a root cause for the discrepancy between TPS calculated and measured lateral profile. However, a potential effect of detector size in terms of two‐dimensional scatter and additional range pull back due to non‐water equivalent materials around each detector needs to be further expanded on and investigated. The complexity of detector size effect will be further investigated.

## 4 DISCUSSION

In this study, we have presented an in‐depth analysis into the QA process at our institute. Overall, the QA process at our institute is quite accurate. Only 6% of beams that undergo QA testing do not pass the 95% 3%/3 mm gamma criterion set out by the center. However, it remains important to understand what is causing the deviations within these 6%, so we can ensure that the accurate dose calculations are provided by TPS and precise delivery of the plan is achieved by the beam delivery system. In the preceding analysis, three main causes of sub‐optimal QA results became apparent: setup error, depth requests that lie in high gradient areas, and the limitations of dose detection system such as a finite size of detector and the spacing between neighbor detectors. The first two causes can be handled using a 2D/3D γ‐index analysis toolkit; such as the one described in this manuscript, that includes comparison points in the proximal/distal dimension along the depth of beam path. This can control for setup error where gross deviations are present or in cases where a fraction of the measurement is in a high‐dose gradient area and only slight deviations are found. However, allowing a search criterion of 3 mm with respect to actual measurement depth may be too loose to detect actual errors occurring during the dose delivery or large deviations of TPS dose calculations. Using 3 mm radius in depth for the 3D γ‐index analysis on measurements using 24 pin‐point chambers in 3D configuration as described in Li et al,^19^ the effects of metals within detector and the plastic block holding detector could be ignored to generate the QA 3D dose distributions in water. With this loose criteria, large deviation of carbon‐ion spot size modeled in TPS was not detected during the PBS‐QA measurements.

The third cause is much more difficult to solve on an immediate basis and warrants a discussion of possible solutions. The main issue with the detection system and the cause for the response are the overall size in the transverse plane. While the volume and the height are small, the sacrifice is made by having detector diameters of almost 5 mm in the transverse plane. In the case of PBS, especially in high energy cases, this detector diameter can be almost twice that of the nominal spot size. Therefore, when the planned dose uses these spots to build up a sharp dose gradient, it can lead to volume averaging and varied responses within detectors that fall in these gradient areas. Taken together, even in the case of accurate setup and lack of dose gradients with respect to depth, the QA test can still fail due to limitations in detector technology. At this point, we cannot correct for errors due to this issue but recognize and note them as they arise.

**FIGURE 11:**
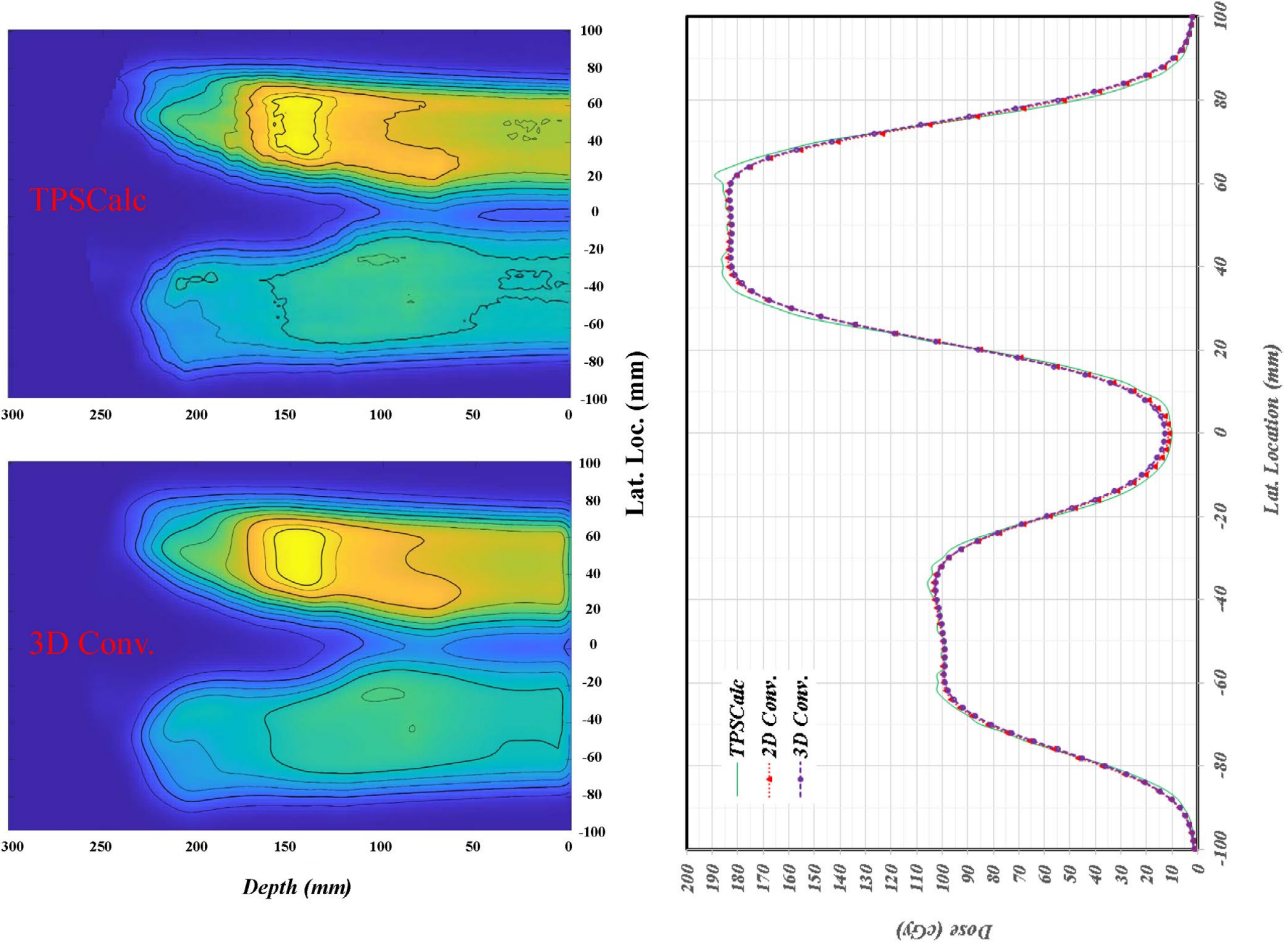
Comparison of convolved measurements with TPS calculated dose. **Left**: TPSCalc and three‐dimensional (3D) convolved dose distribution in the top and bottom panels, respectively. The corners at each side of plateaus are smoothed with lower dose, while the lower doses of valley are filled in with higher doses. **Right**: The one‐dimensional lateral profiles of TPSCalc, two‐dimensional (2D), and 3D convolution are shown. The sharp corners at each plateau are reduced while the doses at valley are increased between the TPSCalc and 2D/3D convolution of detector sizes

The attempt to utlize the 1D analytical simulation was first found to mimic a >3% deviation on the valley between two plateaus with >6%/ mm dose gradient. However, the dose graidents for non‐optimized prostate and breast cases were too small (only ~2%/mm) to correspond the obseved difference between the measurement and TPSCalc. As an expansion of this 1D analytical simulation, the 2D and 3D convolution methods were studied recently only on TPSCalc data only as shown in Figure 11. A simple point smearing function over a 9 × 9 × 9 matrix with a sigma of 2 was applied for each calculated pixel was convolved with neighboring pixels. Because the size of each pixel is 2 mm, the actual size of matrix is 18 × 18 × 18 mm with a sigma of 4 mm mimicking the used detector size of 5 mm in radius. For the 2D convolution, the smearing function along the depth was switched off. For 3D convolution, a smearing of 4 mm may be too large along the depth. The left panels of Figure 11 show the 3D convolved lateral profiles with various isodose levels. Overall distribution between TPSCalc and 3D convolved profile is similar except in the large sharp dose gradients on each side of plateau and the valley between two plateaus. To quantify the difference, the 1D profile was extracted and is shown at the right panel of Figure 11. As seen in 2D profile, the changes around the shoulders and in the valley are about 2%–3%. The change is much larger than the 1D analytical simulation in the Figure 10. Therefore, a 2D and/or 3D convolution method needs to be further investigated for the effect of detector size. By removing the effect of detector size, the actual deviation of dose delivery itself as well as the discrepancy induced by TPS calculation can be investigated.

Instead of a software solution on the issue of detector size, the problem of detector size can be solved in a relatively easy fashion using diodes in photon therapy, but this is not a viable option in particle therapy due to the change of response due to the damage by the particle beam and a large variation of dose response between measurement depths at the pristine peak and 2.0 cm proximal to the peak. Currently, there is much research into ways of improving the detection system with a proper measurement process. Some methods involve fiber optics or silicon‐based flat panel,^20^, ^21^ but there is a strong movement toward log file‐based QA. This process utilizes machine log files as a method for patient‐specific QA. Research has shown that information stored in these files is sufficiently accurate to serve as a QA tool in PBS beams.^22^ Although the implementation of a log file QA program at our institute will provide the efficient approach in most of PBS‐QA measurements, discrepancies between TPS calculated and log file reconstructed doses could still occur and would then require actual measurements. Therefore, an investigation into the use of convolution to reduce the effects of detector size will still provide the important data to identify any deviation of beam delivery with respect to the TPS dose calculation.

## 5 CONCLUSIONS

The three root causes for sup‐optimal PBS‐QA measurements are identified as user error, the effect of high depth dose gradient, and the effect of finite detector size. A 2D/3D γ‐index analysis toolkit improved significantly on sub‐optimal pass rate of measurements. However, a criterion of 3 mm in depth may be too loose for detecting the minor deviation of beam delivery or a minor inaccuracy of TPS dose calculation. The analytical simulation allows for an understanding the effect of detector size on measurement. However, the 1D analytical simulation is limited for the actual complex 3D dose distribution. A 2D and/or 3D convolution approach with a simple point spreading function shows the potential to mimic the response variation of detection system. However, the proper convolution function needs to be further investigated. With an understanding and resolution to these root causes, the goals of patient‐specific QA to detect actual deviation of beam delivery and/or to identify the limitation of dose calculation algorithm in used TPS can be directly related to the failure of PBS‐QA measurements.

## CONFLICT OF INTEREST

None.

## AUTHOR CONTRIBUTIONS

Jacob Ricci contributed on the classification of PBS‐QA measurements in the 2D and 3D gamma index analysis. Wen Hsi contributed the design on the whole contents of this manuscript. Zhong Su provided the review and comments on this manuscript, Karl Mund provides the 3D gamma index analysis tools with instructions for the usage of this tool. Robert Dawson contributes the analysis using the smearing of detector size in the section of discussion, Daniel J Indelicato provides the requirement of passing gamma index of PBS‐QA measurements according to the clinical condition on each measurement.

## Data Availability

The data that support the findings of this study are available from the corresponding author upon reasonable request.
